# Stress at School? A Qualitative Study on Illegitimate Tasks during Teacher Training

**DOI:** 10.3389/fpsyg.2016.01410

**Published:** 2016-09-14

**Authors:** Stefanie Faupel, Kathleen Otto, Henning Krug, Maria U. Kottwitz

**Affiliations:** Work and Organizational Psychology, Faculty of Psychology, Philipp University of MarburgMarburg, Germany

**Keywords:** illegitimate tasks, stress-as-offense-to-self, teaching trainee, qualitative content analysis, social context

## Abstract

What do I expect when stating that “I am going to be a teacher”? Social roles, including professional roles, often become part of people's identity and thus, of the self. As people typically strive for maintaining a positive sense of self, threats to one's role identity are likely to induce stress. In line with these considerations, Semmer et al. recently (e.g., Semmer et al., 2007, 2015) introduced “illegitimate tasks” as a new concept of stressors. Illegitimate tasks, which are defined as *unnecessary* or *unreasonable* tasks, threaten the self because they signal a lack of appreciation regarding one's professional role. Teacher training is a phase of role transition in which the occurrence of illegitimate tasks becomes likely. A holistic understanding of these tasks, however, has been missing up to now. Is there already a professional role identity during teacher training that is vulnerable to threats like the illegitimacy of tasks? What are typical illegitimate tasks in the context of teacher training? In order to close this research gap, 39 situations taken from 16 interviews with teaching trainees were analyzed in the present study on the basis of qualitative content analysis. Seminars and standing in to hold lessons for other teachers were identified as most prevalent illegitimate tasks. More specifically, unnecessary tasks could be classified as sub challenging, inefficient and lacking in organization (e.g., writing reports about workshops no one will ever read). Unreasonable tasks appeared overextending, fell outside responsibility, and lacked supervisory support. Training interventions focusing upon task design and supervisory behavior are suggested for improvement.

## Introduction

Being a teaching trainee can be a difficult and stressful time. Negative health consequences, such as the development of burnout, have already been linked to the teacher training curriculum (e.g., Zimmermann et al., 2012). Similarly, stress and impaired health among teachers in general are prevalent. Stress-related disorders are observed more frequently in the teaching profession than in other professions and are a frequent cause for early retirement in this group (Lederer et al., 2003; Schaarschmidt and Kieschke, 2007). Theoretically based on the stress-as-offense-to-self-concept (SOS; Semmer et al., 2007), this study investigates a potential antecedent, namely teaching trainees' perception of having to execute tasks that should not be expected from them given their professional status. The perceptions that these allegedly *illegitimate tasks* are below (or above) professional status can be demeaning and contradictory to the self-esteem of a teaching trainee and hence induce stress. Illegitimate tasks are *unnecessary* when they lack sense because of poor organization, previous mistakes, or lack of importance. They are *unreasonable* when they lie outside a person's responsibility considering the professional role. In the present study, relevant task characteristics, framework and social conditions of these illegitimate tasks should be identified in order to gain an in-depth understanding of these potentially stress-inducing tasks and provide new information for the reduction of stress and strain during the teaching trainee curriculum—a phase of role transition.

Negative health consequences for teachers and teaching trainees in particular are prevalent (Bauer et al., 2006; Unterbrink et al., 2007; Zimmermann et al., 2012). In a recent study, a comparison of teachers with other professions revealed that female teachers perceive a lower ability to work until retirement and show more psychological fatigue compared to women in other professions (Van Droogenbroeck and Spruyt, 2016). According to the German Federal Statistical Office, 13% of teachers retired early in 2013 due to occupational disability (German Federal Statistical Office, 2014). In line with this, Weber et al. (2005) revealed psychiatric and psychosomatic disorders as the most frequent causes for early retirement among school principals. Comparably, Zimmermann et al. (2012) showed that 44% of teaching trainees in a German sample reported impaired mental health and approximately half of the participants did not feel well-prepared for a teaching career. Similarly, Klusmann et al. (2012) investigated German teaching trainees and found an increase in emotional exhaustion in the course of 9 months. Consistent with these results, Goddard et al. (2006) revealed an increase in the three burnout dimensions (emotional exhaustion, depersonalization, and reduced personal accomplishment) in a sample of young teachers in Queensland who had graduated from university within the past 2 years. The absolute burnout levels exceeded those of a normative reference group. Moreover, participants consistently reported declined role clarity over the course of the 21-month measurement period in this study, which can be explained by teaching trainees' intermediate position during their curriculum.

To fully understand the context of teaching trainees, one has to take a closer look at the structure of the teaching trainee curriculum in Germany. Earning a teaching degree in Germany is a two-fold process. The first stage ends after approximately 5 years of studying with the First State Examination, including both written and oral examinations and a thesis (university degree). During the second stage, a prospective teacher is paid a trainee's salary, expected to work as a teaching trainee while also continuing studies. The second stage ends with the Second State Examination. Prospective teachers can be qualified to teach at different types of schools, such as primary or secondary school. Both structure and content may vary to some extent depending on the school type and across the 16 states of the Federal Republic of Germany. The second stage of the curriculum, which includes teaching in the classroom, takes one and a half to 2 years. It is divided into two main aspects. Firstly, teaching trainees spend a lot of time at school, attending other teachers' classes or teaching classes themselves, partly under supervision. It is important to note that preparation at home, evaluation and critical reflection of lessons belong to the job as well. Aside from teaching, the teaching trainees also take part in school activities and take responsibility for school related tasks, ranging from supervision in school breaks to counseling pupils. Stand-in classes are supposed to be taught only to a certain extent or on a voluntarily basis. In short, during the curriculum teaching trainees become a crucial part of the school staff. They accomplish the same tasks as full time teachers and meet them on an equal footing. In addition to the tasks that full time teachers are required to do, trainees are supposed to reflect on their lessons regularly. For this purpose, they get advice from supervisors and their lessons are graded from time to time. The second main aspect of the curriculum is the future teachers' participation in study seminars for one to one and a half days. The seminars provide a deepening subject-specific and interdisciplinary didactic education. There, the trainees prepare fictional lessons and worksheets, hold presentations and share their teaching experiences. The total curriculum is commonly split up into three parts: The *introduction phase* primarily consists of attending other teachers' classes. During the *main phase* teaching trainees teach lessons autonomously and under supervision but still sit in on other teachers' lessons. The *examination phase* equals the main phase only that teaching lessons are reduced, allowing the teaching trainees to prepare for their Second State Examination, which constitutes the completion of their training. Throughout the course of teacher training, lesson investigations are conducted periodically, where trainees' performances in designing a school lesson are evaluated and graded. Thus, teaching trainees face a challenging dilemma: On the one hand, they take over the role as full teachers. On the other hand, they are still studying and are not autonomous in their decisions yet. Especially when trainees are teaching a class on their own they are treated as equals by other teachers. Then again, lessons are evaluated and graded by these same teachers on a regular basis, putting trainees in a subordinate position.

Beginning teachers are in a transition phase from trainee to professional, which also means a transition into a fully appreciated member of a work group (Elfering et al., 2007). They have to position themselves in the school environment and make choices based on personal evaluations despite many regulations (Coldron and Smith, 1999). Struggling with classroom discipline, motivation and assessment of pupils, and dealing with parents are common challenges (Veenman, 1984). Responsibility increases rapidly (Elfering et al., 2007) and trainees begin to form their professional role: They adopt personal teaching styles, create relationship patterns with pupils, colleagues and supervisors, and develop general principles that serve as guidance for actions and help mastering new challenges (Coldron and Smith, 1999). In contrast to this high amount of responsibility and freedom to teach, trainees are graded and evaluated regularly in examination lessons and must obey to what other teachers or instructors want in certain situations (Flores and Day, 2006). Hence, they constantly switch from their role as a full teacher to their role as a trainee and vice versa, which explains resulting role-related tensions, such as declined clarity (Goddard et al., 2006) or even role ambiguity (Pithers and Soden, 1998). In line with this, Pillen et al. (2013) identified several professional identity tensions beginning teachers typically experience. For example, many teaching trainees were confronted with ideas and methods on how to learn teaching that contradicted their own ideas. They also felt dependent on mentors or colleagues, which conflicted with their goals to apply own teaching styles. Moreover, the trainees reported that expectations of them as teachers were too high, which made them sometimes feel like students. In other situations, however, they were willing to take responsibility but were still treated like students. Trainees who are confronted with conflicting expectations have to choose which side to take and are therefore feeling role uncertainty (e.g., Wirtz et al., 2013). In sum, several authors (e.g., Goddard et al., 2006; Pillen et al., 2013) showed that the teaching trainee curriculum is a phase of transition, in which a professional identity of teaching trainees is developing, but not yet clearly defined for trainees themselves, but also for teachers, parents or pupils. Importantly, it is this ambiguity in particular that makes the curriculum an interesting and relevant context for the analysis of illegitimate tasks, since it increases the risk of their occurrence.

Now, why do illegitimate tasks matter in the teaching trainee curriculum? The answer to that is the self. People strive toward a positive feeling of self-worth (Thoits, 1991; Tesser, 2000; Epstein, 2006; Sedikides and Gregg, 2008). Being an accepted member of a social group is a strong human motivation (Baumeister and Leary, 1995) that is closely related to a person's self-esteem (Gruenewald et al., 2004; Leary, 2005). Moreover, the professional role is of importance for most people and represents a significant portion of the self (Stryker and Burke, 2000; Sluss and Ashforth, 2007). Hence, when an individual is treated disrespectfully because a subjectively illegitimate task has to be accomplished, the individual does not feel as an appreciated member of the work group. It is important to note that it is not the task itself, but the social message transmitted through the task that is crucial. Subjectively *unnecessary* or *unreasonable* tasks (e.g., Semmer et al., 2007) are interpreted as degradation for one's professional role identity: The person who assigned them, most likely a supervisor, must lack professional respect or else he or she would not have made the request. Tasks classify as *unnecessary* when they are perceived as senseless because they are poorly organized, previous mistakes were made or the task itself lacks importance. Creating documents that are hardly read by anyone constitutes such a task (Semmer et al., 2013). In the context of training teachers, this should not be demanded of a trainee because the task has no value and is avoidable. Thus, it poses a threat to the trainee's professional role. Teaching trainees in Pillen et al. (2013) reported feeling pushed to accomplish many additional tasks although they wanted to spend more time on teaching, which can cautiously be interpreted as a first hint on the existence of unnecessary tasks during teacher training.

For the second dimension, *unreasonable* activities, the teaching trainees' concept of their professional role is of great importance. By definition, these tasks exceed the level of responsibility that could normally be expected of an employee considering his or her status in the profession. Specifically, a task is perceived unreasonable when it falls outside the scope of the professional role in a given context. Moreover, tasks not in accordance with the experience or expertise level of an employee potentially create illegitimacy as well (Semmer et al., 2007). Hence, for an experienced full time teacher it can be perfectly legitimate to stand in for a sick colleague on short notice. However, a teaching trainee, who is new to the job and needs more preparation time to conduct a lesson, might perceive the same task as unreasonable because it falls out of his or her responsibility with respect to the newcomer position. Unreasonable tasks are assumed to be of relevance during teacher training. As described previously, roles are not always clearly defined during this phase of transition. Therefore, it is possible that the perceptions differ among trainees, supervisors and colleagues of whether the task is legit considering the trainee's professional status. The relevance of illegitimate tasks has been shown in studies with several psychological indicators for health and well-being (Stocker et al., 2010; Björk et al., 2013; Semmer et al., 2015), sleep quality (Pereira et al., 2014), and cortisol levels (Kottwitz et al., 2013). However, despite these results, research concerning illegitimate tasks is incomplete up till now. First, a holistic understanding of these tasks is missing. How are they constituted? In which situations do they occur? How do they relate to professional role perceptions? Certainly, answers to these questions are of special value not only in the SOS research framework but also in terms of practical considerations because only an in-depth understanding of illegitimate tasks can help the prevention of negative health consequences associated with them. Second, illegitimate tasks have never been analyzed in the context of teaching trainees so far. However, especially the teaching trainee curriculum is a phase of professional role transition for the prospective teachers, and thus, assumed to be a likely source of illegitimacy. Consequently, the curriculum is an interesting and relevant research context for illegitimate tasks.

Accordingly, the present study aims to answer the following research questions:

(1) How do teaching trainees define their professional role? (2) Which tasks are perceived as illegitimate during teacher training? (3) How are these tasks constituted, what are related framework and social conditions, and who is made responsible?

## Methodology

### Data collection

#### Sample

In total, 24 teaching trainees who were completing their training at secondary schools from two states in Germany were interviewed for the study. The first trainees were approached by contacting schools and study seminars, and the following ones were recruited via word-to-mouth recommendation. Elementary school trainees were not included due to different work and curriculum structure. Participation was voluntary and informed consent was given prior to each interview. Eight interviews were excluded from the analysis in the present study, because participants reported that they were never assigned any illegitimate tasks during the curriculum. Accordingly, 16 interviews with teaching trainees (6 males, 10 females) with ages ranging from 24 to 30 years (*M* = 27.5, *SD* = 2.03) were included in the analysis. All but one participant were in the main phase of their curriculum (one in the examination phase). The average amount of teaching at school was 55.4 lessons monthly (one lesson lasts 45 min) while the average time spent in study seminars was 24.1 h monthly. All but two participants taught at grammar schools (German Gymnasiums). Two participants taught at comprehensive schools. In total, 11 out of 16 participants rated being a teacher as their desired profession.

#### Content of interview guideline

A semi-structured interview guideline with a total of six open questions was used for qualitative information collection. This guaranteed intersubjective comparability (Lamnek, 2010) and nevertheless, gave freedom in formulations plus offered the opportunity to ask further in-depth questions if necessary (Hopf, 2013). The first two questions addressed teaching trainees' professional role definition. Specifically, they asked for typical role matching and not role matching tasks in the curriculum (How would you define your role as a teaching trainee? What are tasks that you include in your role? What are tasks that you don't include? Please describe your understanding of your role at school and in the study seminar.). The third question was whether the trainee ever felt unappreciated during the curriculum and trainees were asked for a description of the situation. Interviews were stopped if no such situation was reported. If a situation was described, two questions specifically addressing illegitimate tasks followed. One related to unnecessary tasks and reads, “Have you already been given a task or experienced a situation during the curriculum—either at school or in the study seminar—which you perceived as unnecessary?” and one related to unreasonable tasks: “Have you already been given a task during the curriculum—either at school or in the study seminar—that should have been conducted by someone else?” Teaching trainees were asked to describe the situations and further in-depth questions relating to reasons for the perceived illegitimacy, framework, and social conditions and involved persons followed. Finally, the trainees were encouraged to make suggestions for improvement of the reported situations. Demographic data (see sample section) were collected at the end of each interview.

### Data analysis approach

Qualitative content analysis following Mayring (2015) was chosen as an adequate method for analysis. The strength of this procedure is that it follows a predefined process scheme. Analysis is strictly rule guided and continuous revisions are made in return loops. All these features guarantee a high degree of transparency, procedures are comprehensive and results are reproducible. Finally, intercoder reliability provides a further quality criterion (Hopf, 2013; Mayring, 2015). Central element of analysis is a category system that contains and structures all relevant information. In the present study, the computer software MAXQDA 10 (VERBI GmbH, 2013) served as a tool for the creation of categories and was used for the analysis of the interviews.

For a definition of the *professional role*, which constitutes the first major dimension in the category system, it was differentiated whether trainees defined themselves as teachers or trainees and *role matching* vs. *not role matching* tasks were distinguished according to research on illegitimate tasks (e.g., Semmer et al., 2015) and professional identity development in the teaching profession (e.g., Coldron and Smith, 1999; Pillen et al., 2013). Based on the curriculum structure and the two-dimensionality of illegitimate tasks (Semmer et al., 2007) the second major dimension *illegitimate task* was further divided in terms of task type and context (school vs. study seminar). Correspondingly, four structuring dimensions resulted: *Unnecessary task at study seminar, unreasonable task at study seminar, unnecessary task at school*, and *unreasonable task at school*. Whenever this distinction was not possible, it was coded into a fifth structuring dimension, namely *other illegitimate task.* For further characterization of illegitimate tasks, the main categories *the activity, task characteristics, framework conditions, social conditions*, and *person responsible* were created with reference to theoretical assumptions and research on illegitimate tasks (e.g., Semmer et al., 2007, 2015) and typically experienced tensions among teaching trainees (Veenman, 1984; Pillen et al., 2013). While social conditions can be understood as an umbrella term for categories that referred to direct social interaction, framework conditions referred to more structural elements that cannot be changed easily in a given situation (e.g., guidelines). After accomplishing a first theoretically guided draft of the category system, going through the material repeatedly resulted in further extension of the categories.

Coding of all interviews by a second rater revealed very good or good overlap in codings with reference to Wirtz and Caspar (2002): Cohen's Kappa values for all but one main category were >0.70 (*social conditions* for unnecessary tasks at school *K* = 0.66). Two subcategories, namely *lack of appreciation by a supervisor* during unnecessary tasks at school (*K* = 0.48) and *dependence on supervisor* during unreasonable tasks at school (*K* = 0.48) had lower Kappa values but were not excluded from the analysis due to recommendations by Wirtz and Caspar (2002) that values ranging from 0.40 to 0.60 can be tolerated.

## Results

To begin with, results addressing the first research question (how do teaching trainees define their professional role) are presented. Subsequently, results addressing the second and third research question that are related to illegitimate tasks are outlined: Which tasks are perceived as illegitimate during teacher training? How are these tasks constituted, what are related framework and social conditions, and who is made responsible?

### Teaching trainees' professional role perception

While some trainees described their role as a student (“Regarding teaching you are still in training and someone always supervises you.”) others already defined themselves as full teachers by stating, “The truth is that you are a full teacher […].” Consistent with these differences in role perception, trainees had different perspectives whether certain tasks are matching their professional role or not. Tasks typically reported by teaching trainees from their daily routine are illustrated by a pointed statement from one trainee who said, “I have to teach, I have to educate, I have to advise, and thus, also talk to parents.” Moreover, personal development and reflecting on one's role as a teacher were considered important aspects of the curriculum, showing in statements such as “develop in my teacher role.” As for these reported “core tasks,” trainees agreed that administrative or cleaning tasks do not correspond to a trainee's role, but also not necessarily to a teacher's role. As one trainee stated, “Not included is every task that normal teachers should also not have to do. Although, I think there are teachers who are willing to exploit themselves […].”

It is particularly interesting that there was disagreement whether participation in examinations or conferences, standing in for colleagues and supervision in school breaks are tasks corresponding to the role as a teaching trainee. Furthermore, some trainees perceived extra duties at school such as “offer[ing] a working group or […] [being] active in school life in general” as part of their teacher training while others reported these kinds of activities as extra role tasks. Many trainees perceived the study seminar, which constitutes a central element in the German teaching trainee curriculum, as ancillary (“I think teaching is a typical task. The seminar is somewhat secondary.”).

### Perceived illegitimate tasks during teacher training

From a total of 39 reported situations relating to illegitimate tasks, 15 related to the study seminar (12 unnecessary, 3 unreasonable), 19 referred to situations at school (7 unnecessary, 12 unreasonable) and five situations could not be classified in terms of task type and context and fell into the structuring dimension *other illegitimate task*.

It was striking that the study seminar as a core structural element of the German teaching trainee curriculum was often described as unnecessary and thus illegitimate (see Table 1).

**Table 1:** **Illegitimate activities in the school and study seminar context**.

**Categories**	***N* (%)**	***N* (%)**	**Example**
**Context study seminar**	**Unnecessary**	**Unreasonable**	
**M4.:The activity**	**14 (37)**	**2 (5)**	
S4.1:administrative task			
S4.2:attendance and contribution at seminar	14 (37)	2 (5)	Now and then, there are situations in the study seminar that lack sense (laughs).
S4.3:performance assessment^*^			
S4.4:mastering of a problem situation^*^			
S4.5:substitution^*^			
S4.6:participation at conferences^*^			
S4.7:school development, extra duty^*^			
**Context school**	**Unnecessary**	**Unreasonable**	
**M4.:The activity**	**9 (24)**	**13 (34)**	
S4.1:administrative task	3 (8)	1 (3)	In every meeting at school teaching trainees are asked to keep the minutes.
S4.2:attendance and contribution at seminar	3 (8)		Sometimes these seminars at school that we have [are unnecessary].
S4.3:performance assessment^*^		2 (5)	I still have problems to grade and design examinations. But the job as teacher requires it.
S4.4:mastering of a problem situation^*^		4 (11)	One child has social-emotional special needs and once got mad. He was flailing arms and shouting that he was going to kill everyone.
S4.5:substitution^*^		6 (15)	On average, teaching trainees are supposed to stand in holding lessons for other teachers approximately three to 4 h. In February, I had eleven.
S4.6:participation at conferences^*^	1 (3)		It is required to participate because it is part of the teacher job. But characteristically, teachers talk a lot and it takes so much time.
S4.7:school development, extra duty^*^	2 (5)		We always get the impression […] that it is necessary to engage in extracurricular activities to get a good grade from the school principal.
**Type of task**	**23 (61)**	**15 (39)**	
**Total**	**38 (100)**		

M, Main category; S, Sub category; N, Frequency. (%), Frequency of coding in percentage (38 codings = 100%). Total N: 38 codings from 39 situations. For each situation a subcategory was only coded once.

* These categories only apply to situations at school but not to situations in the study seminar.

For example, a trainee stated, “Now and then, there are situations in the study seminar that lack sense (laughs).” In these situations, trainees often reported that the content of the seminar was *sub challenging* (“We all studied and learned these things at university.”) or *not relevant for later practice* as one trainee said: “I am sure that in 80% of the cases I don't need these things.” Aside from having the feeling *not to learn something* (“You don't have the impression to learn anything valuable that helps you as teaching trainee.”), another major problem were *inefficiencies* in the seminar organization as described by one trainee: “We talked about the subject for more than 30 min […]. It was dragged on too long.” A possible reason why illegitimacy in terms of unnecessary tasks often occurred in the study seminar was given in explanations by trainees emphasizing the *heavy workload* during the curriculum. For example, one trainee reported that “parallel to all the other tasks such as lesson investigations, teaching, and so forth it is just a great deal of work.” Moreover, *lack of planning and organization* (“I would have considered it more useful if that was done beforehand.”) and inexperienced *instructors* (“It's the first time that my instructor holds [the seminar]. Maybe she lacks experience.”) also seemed to be relevant in this context. For an overview of all characteristics and frequency distributions of unnecessary tasks in the study seminar see Table 2.

**Table 2:** **Characteristics of illegitimate tasks**.

	**Context study seminar**		**Context school**		
	**Unnecessary**	**Unreasonable**	**Unnecessary**	**Unreasonable**	**Example**
**Category**	***N*** **(%)**	***N*** **(%)**	***N*** **(%)**	***N*** **(%)**	
**M5:Task characteristics**	**36 (24.2)**	**6 (4.0)**	**15 (10.2)**	**17 (11.4)**	
S5.1:unpopular				2 (1.3)	They tell you to carry out the task “en passant.” And no teacher likes standing in for another teacher.
S5.2:senseless	13 (8.7)	2 (1.3)	7 (4.7)	1 (0.7)	And I didn't see how it made sense.
S5.3:not educational	4 (2.7)	1 (0.7)	1 (0.7)		You don't have the impression to learn anything valuable that helps you as teaching trainee.
S5.4:sub challenging, redundant	9 (6.0)		1 (0.7)		We all studied and learned these things at university.
S5.5:overextending	1 (0.7)		1 (0.7)	8 (5.4)	
S5.5a:quantitatively	1 (0.7)			2 (1.3)	In my opinion, a work load of 11 h was just too high for a trainee.
S5.5b:qualitatively			1 (0.7)	6 (4.0)	Any other teacher who knew the subject could have done a better job than me […] having no clue what they are doing.
S5.6:not practically relevant	3 (2.0)	1 (0.7)	1 (0.7)		I am sure that in 80% of the cases I don't need these things.
S5.7:inefficient	6 (4.0)		4 (2.7)		We talked about the subject for more than 30 min […]. It was dragged on too long.
S5.8:outside responsibility area		2 (1.3)		6 (4.0)	I absolutely don't see that as my job. She earns some big bucks (laughs) by sitting there and letting us work.
**M6:Framework conditions**	**13 (8.7)**	**7 (4.7)**	**6 (4.0)**	**5 (3.3)**	
S6.1:lack of planning and organization	2 (1.3)	1 (0.7)	3 (2.0)	2 (1.3)	I would have considered it more useful if that was done beforehand.
S6.2:additional effort through poor work of others	3 (2.0)	2 (1.3)			I think it's not enough to distribute topics […]. I expect more from the instructor.
S6.3:guidelines	3 (2.0)	3 (2.0)	2 (1.3)	2 (1.3)	
S6.3a:lacking flexibility	2 (1.3)	1 (0.7)	2 (1.3)		We went to some kind of youth hostel and were required to sleep there. […] That was strange for people who lived nearby, for example.
S6.3b:lacking uniformity, arbitrariness	1 (0.7)	2 (1.3)		2 (1.3)	There's an agreement that [these meetings] shouldn't be too long. I think no one cares about it.
S6.4:heavy workload	5 (3.4)	1 (0.7)	1 (0.7)	1 (0.7)	Parallel to all the other tasks such as lesson investigations, teaching, and so forth it is just a great deal of work.
**M7:Social conditions**	**2 (1.3)**		**6 (4.0)**	**19 (12.7)**	
S7.1:disrespectful treatment			2 (1.3)	4 (2.7)	
S7.1a:by supervisor			2 (1.3)	3 (2.0)	You are treated disrespectfully by some teachers because they think of you as the damn trainee and they put themselves above you.
S7.1b:by parents				1 (0.7)	You are in the role of being a trainee and being new […]. And—in this case the parents—let you feel this.
S7.1c:by pupils					
S7.2:lacking social support from supervisor			1 (0.7)	6 (4.0)	I did not know the child at all, […] no one informed me. In this moment I thought it would be great if the teacher was here.
S7.3:dependence on supervisor			2 (1.3)	3 (2.0)	Just because the school principal writes a report […] which counts into the final grade.
S7.4:lacking information, communication and cooperation	2 (1.3)		1 (0.7)	6 (4.0)	If I had been informed about the problem earlier, it would have been easier to react in the situation.
**Category**	***N*** **(%)**	***N*** **(%)**	***N*** **(%)**	***N*** **(%)**	
**M8:Responsible person**	**5 (3.4)**	**2 (1.3)**	**2 (1.4)**	**8 (5.4)**	
S8.1:teacher, principal, mentor			1 (0.7)	8 (5.4)	It would have been the teacher's responsibility.
S8.2:instructor/person in charge for the apprenticeship	5 (3.4)	2 (1.3)	1 (0.7)		The core instructor leaned back and watched.
**Type of Task**	**56 (37.6)**	**15 (10.1)**	**29 (19.5)**	**49 (32.8)**	
**Context**	**71 (47.7)**		**78 (52.3)**		
**Total**	**149 (100)**				

M, Main category; S, Sub category. N = 149 codings from 39 situations. (%): Frequency of coding in percentage (149 codings = 100%). For each situation a subcategory was only coded once.

While many situations in the study seminar were reported as unnecessary, for some situations illegitimacy was also perceived because the seminar was unreasonable, and thus went beyond trainees' responsibility (“I have to prepare a workshop for a program, […] however, in my opinion, it isn't my job to do this.”). As the quote shows, this dimension of illegitimacy was perceived when instructors delegated tasks to trainees that they perceived as an additional effort. In line with this, one trainee for example described, “I think it's not enough to distribute topics […]. I expect more from the instructor.”

Although not directly asked in the interview questions, associations were made by trainees between illegitimacy and stress. For example, one trainee stated, “I see it as burden that you have to deliver that much in the study seminar.” Another trainee clearly differentiated, “I think, my feelings are not due to bad quality work by the instructors but rather due to the enormous stress I'm under.” A threat to the self as core element of the SOS-concept could be observed for a trainee who reported an illegitimate task and stated, “I felt like a fool (laughs).”

Referring to the school context, *administrative tasks* such as keeping the minutes were listed as unnecessary and unreasonable (“In every meeting at school teaching trainees are asked to keep the minutes.”). Moreover, study participants identified some situations as unnecessary including *attendance at seminars* at school, the *participation at conferences*, and tasks related to *school development and extra duties* (see Table 1). Referring to extra duties at school, one trainee described, “We always get the impression […] that it is necessary to engage in extracurricular activities to get a good grade from the school principal.” In this context, the trainee also perceived a lack of respect (“almost disrespectful because no one knows what one does additionally on a voluntary basis apart from the school context, for example”). As for the study seminar, *lack of planning and organization* also constituted a problem related to unnecessary tasks at school (“[The program] had just started and was organized pretty poorly”). Moreover, *inefficiencies* were reported in the context of unnecessary tasks at school similar to unnecessary situations in the study seminar.

Compared to the study seminar, at school more situations were perceived as illegitimate because they were unreasonable. Thus, at school a major problem seems to be overstepping responsibilities, whereas lack of sense seems to be more prevalent in the study seminar. Tasks that were associated with illegitimacy in terms of unreasonableness at school are *performance assessment, substitution lessons*, and *administrative tasks* (Table 1). Moreover, the *mastering of problematic situations* evolved as a category which describes special school situations in which teaching trainees had to deal with classroom conflicts or were confronted with family conflicts or behavioral problems of pupils. As the description shows, these situations had an individual, exceptional character and were consistently perceived as unreasonable (see Table 1). A trainee described one situation that fell into this category: “[…] I was on my own. […] One child has social-emotional special needs and once got mad. He was flailing arms and shouting that he was going to kill everyone.” Another trainee reported: “I had a pupil in a senior class who was bullied by the others because he was allegedly gay. […] The classroom climate was really bad. So I talked to the tutor of the class and again he denied noticing the issue. […] In these situations you really wish for some more support.” As the quotes illustrate, unreasonable tasks at school were often described as *outside the trainee's responsibility area.* They were *overextending*, with *qualitative* compared to *quantitative overextension* as a frequent problem. One trainee reported for example, “Any other teacher who knew the subject could have done a better job than me […] having no clue what they are doing.” Furthermore, *unpopular* was another attribute that characterized unreasonable tasks at school. As an example, one trainee described, “They tell you to carry out the task “en passant.” And no teacher likes standing in for another teacher.”

While additional effort because of poor work of others did not constitute a problem in the context of unreasonable tasks at school, *lack of planning and organization* and *heavy workload* were identified as problems, similar to situations in the study seminar (also see Table 2). *One* trainee for example described a situation where he had to stand in for a colleague on short notice because of lacking organization, “Last week I was told on Monday that I have to stand in the first two lessons on Tuesday.” Finally, *arbitrary or not unique guidelines* were associated with unreasonable tasks at school as well (“There's an agreement that [these meetings] shouldn't be too long. I think no one cares about it.”). As one trainee said, teachers were hold accountable in these situations: “It would have been the teacher's responsibility.” or “The other teachers could also do that.”

An interesting pattern appeared, shifting the focus on social aspects that are associated with illegitimacy. It is striking that social problems were almost never reported with reference to situations in the study seminar but were of great relevance at school (see Table 2). As some earlier descriptions of situations at school already suggest, illegitimate tasks at school were especially associated with *lacking social support from a supervisor* as became clear in descriptions such as, “I did not know the child at all, […] no one informed me. In this moment I thought it would be great if the teacher was here.” Moreover, illegitimacy in this context was often associated with lacking *information, communication*, and *cooperation*. Illustrating this, a trainee stated, “There was no chance or time to consult with the expert colleagues and the class teacher […] to jointly think about the situation and come up with some useful measures,” whereas another trainee similarly reported, “If I had been informed about the problem earlier, it would have been easier to react in the situation.”

Moreover, *disrespectful treatment by a supervisor* or *by parents* was described. Teaching trainees reported situations “where you notice that the teachers think the younger ones should do that.” Another statement by a trainee that described a situation at school was, “You are treated disrespectfully by some teachers because they think of you as the damn trainee and they put themselves above you.” A description about an interaction with parents was: “You are in the role of being a trainee and being new […]. And in this case the parents let you feel this.” *Dependence on a supervisor* evolved as another category associated with illegitimacy at school and was characterized by statements such as, “Just because the school principal writes a report […] which counts into the final grade.”

Comparable to the study seminar, situations of illegitimacy were again associated with feelings of stress or a threat to the self by teaching trainees. For example, trainees reported to be “overwhelmed” or “stressed” by the task. One trainee also described a situation where she “felt like a fool” because a teacher persuaded her of standing in for him. Similarly, another trainee reported, “During the conversation [with a teacher and the parents of a student], I got the impression that I had to justify myself for talking to the student about his behavior […].”

In situations where a classification into task type or context was not possible it was coded into the structuring dimension *other illegitimate task*. Results in this dimension are very similar to task and context specific results, and thus remain unattended here. An exception occurred in two situations at school where trainees described situations as illegitimate, and illegitimacy was associated with *disrespectful treatment by pupils*. One trainee was assigned the task to teach a class on how to handle a computer program and “got feedback from pupils that one should prepare better.” Another trainee reported a situation at school: “I was trying to address their social behavior but they didn't accept what I said.” Other than this, no major difference from context and task specific results occurred.

## Discussion

### Interpretation of results

The aim of the present study was to gain an understanding of how teaching trainees define their professional role. In relation to that, illegitimate tasks and typical task characteristics during the curriculum should be identified. Moreover, associated framework and social conditions and responsible persons ought to be revealed.

Our study results confirm existing research concerning teaching trainees' (professional) role. Teaching trainees in the present study had different conceptions about whether they adopt a role as a teacher or as a trainee, which highlights their intermediate and partly unclear role (e.g., Pithers and Soden, 1998; Elfering et al., 2007). Consistent with this, there were many tasks (central examinations, supervision, conferences, seminar lectures, substitution lessons, and school development and extra duties) that were reported as role confirming by some trainees but as extra role tasks by others. It is striking that illegitimacy was especially reported in the context of these tasks but not the reported “core tasks” (teaching, supporting, counseling and educating pupils and personal development). This also confirms existing research stating that illegitimacy mainly occurs within ancillary but not within core tasks (Semmer et al., 2015).

Focusing on characteristics of illegitimate tasks, several important new insights were gained. First, it is striking that there were major differences in the results depending on the situational context in which illegitimacy occurred. For example, while unnecessary tasks were the dominant problem in the study seminar because it was the seminar itself that was perceived as unnecessary (see Table 1), unreasonableness was more prevalent in the school context. Even more important however, it was the context that determined whether social conditions constituted a characteristic of illegitimacy but not the task or task type itself. More specifically, certain aspects could not be classified as unique characteristics for an unnecessary compared to an unreasonable task. Rather, it depended on the context—school vs. study seminar—whether these aspects were of relevance. There is a visible tendency that social aspects are a more prevalent problem in association with unreasonable tasks. However, given the qualitative design of the study and the small number of codings, this result has to be interpreted with caution. Nevertheless, the present study clearly revealed that an analysis of illegitimate tasks should always include the situational context. Otherwise, important aspects remain undetected or are even misinterpreted. Despite this context specificity, the detailed analysis of social aspects associated with illegitimacy was an innovative feature of this study. It added to existing research on illegitimacy by providing concrete information regarding what illegitimacy really means, at least in the context of teaching trainees. As for social aspects, lack of social support by supervisors; disrespectful treatment by supervisors, parents, or pupils; lacking information or cooperation; and dependence on the supervisor are aspects that were associated with illegitimacy, and thus, potentially contribute to a stressful situation. Building on these results, emotional labor is an aspect that deserves further attention in future research. Teaching trainees in the present study reported disrespectful treatment by pupils and by supervisors—situations in which demands for emotional labor are increased: While on the one hand trainees' professional self is threatened, on the other hand showing positive emotions is part of their job. Research on teachers has shown that deep acting (being able to influence one's own emotions in an appropriate manner) is beneficial for health, whereas surface acting is associated with emotional exhaustion (Philipp and Schüpbach, 2010). While appreciating their work is the most important intervention, providing proper training in redefining their tasks might also help to reduce perceived illegitimacy by enabling trainees to engage in deep acting and thus reduce their experienced level of stress. So far, illegitimate tasks have been described as a two dimensional construct consisting of unnecessary and unreasonable tasks (e.g., Semmer et al., 2007). Consistent with these definitions, we found that unnecessary tasks can be distinguished from unreasonable tasks. While unnecessary tasks were related to inefficiencies, this was not the case for unreasonable tasks (see Table 2). In addition to previous findings, we found that unnecessary tasks were reported to be sub challenging or redundant while this did not occur at all for unreasonable tasks. The Bern Illegitimate Tasks Scale (Semmer et al., 2010), which is the typical instrument for measuring illegitimacy, already includes inefficiency but does not account for sub challenge, an aspect that deserves attention nevertheless. This is especially the case because sub challenge (or overextension as the opposite) were aspects by which unnecessary and unreasonable tasks could clearly be distinguished. As already stated, sub challenge was only found in association with unnecessary tasks. In addition, it is important to note that the opposite was found for unreasonable tasks. Specifically, qualitative overextension was something particularly characteristic for unreasonable tasks. Thus, it seems as if we detected another characteristic by which the two dimensions of illegitimate tasks are clearly separable—at least in teacher training—which also speaks on behalf of the two dimensionality of the construct. However, since we already stated the importance of the context other (quantitative) investigations are necessary to confirm this result.

Another interesting finding of the present study addresses framework conditions that are associated with illegitimate tasks. Reports by teaching trainees clearly revealed that guidelines framing a situation potentially contribute to the occurrence of illegitimacy. While no clear pattern was visible whether guidelines are too strict and inflexible or too loose and arbitrary, one conclusion can still be drawn: It is not only the task design itself or the social interaction that determines illegitimacy. Rather, one has to clearly investigate structures that promote or hinder the development of illegitimacy. Thus, by considering given structures scholars may shed light into why illegitimacy occurs in some contexts but not in others. In practical terms, one should be aware of the importance of structural guidelines and their consequences as a first step toward less illegitimacy.

While the definition of illegitimate tasks, the Bern Illegitimate Tasks Scale (Semmer et al., 2010) and some of our results suggest that unnecessary and unreasonable tasks are distinct constructs, we also found unexpected *similarities* between the two constructs. For example, while the definition and the Scale classify *unnecessary* tasks as avoidable by better planning or organization, we found this characteristic for both task types, unnecessary and unreasonable tasks. Hence, results of the present study raise the question whether this aspect embedded in the typical measurement of unnecessary tasks is able to distinguish between the dimensions in teacher training. Rather, reconsideration is recommended whether there are other task characteristics that would offer better separation of the dimensions. The same applies to additional effort that evolves due to mistakes or poor work of others. Following the Bern Illegitimate Tasks Scale, this is a specific feature of unnecessary tasks. However, this could not be confirmed in the present study. Instead, this aspect was something context but not task specific. In particular, poor work of others was described in the context of the study seminar (for unreasonable and unnecessary tasks) (see Table 2) but did not occur at school. Thus, again, our results suggest reconsideration whether the Bern Illegitimate Tasks Scale validly measures unnecessary tasks in the context of teacher training. In line with this, the dimension *other illegitimate task* evolved because the assignment to one category (unnecessary vs. unreasonable) was not always possible. This draws into question whether a separation is always useful (cf. Semmer et al., 2013).

In sum, the results of the present study partly suggest that illegitimate tasks are comprised of two dimensions, since we identified certain task characteristics that allowed a separation. Examples for those characteristics which separated the two task types in our study were being senseless or inefficient or being outside one's responsibility area. However, especially regarding the definition and measurement of *unnecessary tasks* there were aspects that could not serve as distinguishable characteristic for this dimension in the present study. Hence, reconsideration is recommended here. A first approach could be a detailed analysis of how far sub challenge vs. overextension are able to differentiate unnecessary from unreasonable tasks. Moreover, the situational context should be taken into account as clear differences depending on the context evolved in the present study.

### Strengths and limitations

Eight out of 24 teaching trainees had to be excluded from the analysis because they pointed out that the concept of illegitimate tasks did not apply to them. While an exclusion rate of one third seems relatively high, it is otherwise alarming that two thirds of interviewed teaching trainees reported illegitimate tasks. We did not directly ask for links between illegitimate tasks and stress or threats to the self. Questions addressing these issues could have created a closer link to the theoretical framework underlying the study and is recommended for future investigations. Nevertheless, the fact that trainees reported feelings of stress and threats to the self although we did not directly address these elements of the SOS concept in our interview questions, can also be interpreted as an even stronger support for the supposed associations.

In the present study, a comparison of all 24 teaching trainees was not conducted. However, it would have been interesting to reveal individual or organizational aspects that led to the differences in perceived illegitimacy. Björk et al. (2013) already identified the organization as relevant for the experience of illegitimate tasks in a sample of managers. The same could apply to the teaching profession. It might depend on the school or study seminar whether illegitimacy occurs. More research is necessary here to identify specific organizational and individual factors. Moreover, future investigations should test how widespread illegitimate tasks really are among teaching trainees.

Two subcategories (*lack of appreciation by a supervisor* during unnecessary tasks at school and *dependence on supervisor* during unreasonable tasks at school) had lower interrater reliability values (*K* = 0.48) suggesting that these categories were less clear. Lower agreement here could be due to the fact that dependency and appreciation depict rather abstract constructs that would have needed further definition in order to gain unambiguous results. Although we followed inclusion recommendations by Wirtz and Caspar (2002), results relating to these categories have to be interpreted with caution. Illegitimate tasks comprise a complex construct and the perception of illegitimacy is strongly influenced by subjective evaluations. Therefore, an integration of different perspectives (e.g., full teachers, instructors, and pupils) would have been interesting to fully understand the development and preservation of illegitimate tasks in the teaching context. This remains open for future research. Moreover, this study exclusively focused on illegitimate tasks and the SOS concept. For future research, a stronger distinction from related concepts is needed. Especially interactional justice, which includes respectful interactions with authorities (Colquitt, 2001; Cropanzano et al., 2001), should be distinguished. Some studies controlled for organizational justice and found results that speak on behalf of illegitimate tasks as a distinct construct (e.g., Semmer et al., 2010, 2015). However, during interviews with teaching trainees a distinction was difficult at times. The group value model (Tyler, 1989) also contains need of belonging and appreciation in the work context as crucial factors that are relevant for a person's identity and positioning within a group. Similarly, social stressors focus on “social animosities, conflicts with co-workers and supervisors, unfair behavior, and a negative group climate” (Dormann and Zapf, 2002, p. 35) and likewise include self-threatening elements as causes for stress (Holz et al., 2004). Considering these models in the analysis to prove the additional value that illegitimate tasks and the SOS concept offer was not aim of the present study but should be considered in further examinations to underline the uniqueness and relevance of illegitimate tasks.

The application of illegitimate tasks to the teaching trainee curriculum yielded important new insights. Many teaching trainees had experienced such tasks and gave detailed descriptions about corresponding situations which show the concept's relevance. Two strengths of the present study are especially noteworthy: (1) Interviews and qualitative content analysis following Mayring (2015) offered strong methods that allowed an open and unbiased data collection and analysis of the teaching trainee context. It was possible to build on existing research while the identification of in-depth insights was facilitated. (2) The construct of illegitimate tasks is very specific, which makes it easy for people to talk about corresponding situations. To think of a previous unnecessary task is easier than to talk about a rather abstract construct like lacking appreciation. The approach facilitates the detection of an indirect form of lacking appreciation that otherwise would probably remain undetected.

The present study contributes to existing research by revealing typical tasks, characteristics, framework and social conditions, and responsible persons associated with illegitimate tasks during teacher training. Furthermore, teaching trainees' role definitions helped to put illegitimate tasks into context and also contributed to research on role identity development in this group. The present study also demonstrated that teaching trainees experience strain, considering statements such as: “There are a lot of situations where one just feels overloaded or overstrained. […] It is alarming that many people have mental problems during this time or mental breakdowns close to an examination.” Longitudinal designs are necessary to examine whether illegitimate tasks are causally responsible for such reactions. While some results of the present study specifically refer to the teaching trainee curriculum, others are more general and are likely to apply to other professions, offering new opportunities for the examination of illegitimate tasks as a general construct.

### Practical implications

Speaking in practical terms, awareness about ambiguity and tensions that teaching trainees experience during teacher training is a first important step toward less illegitimacy. Teachers, instructors, mentors, and trainees should talk about their roles and clarify reciprocal expectations they have. In doing so, exceeding trainees' responsibilities and under- or overestimation of their skills could be reduced. Moreover, instructors and teachers who assign tasks to teaching trainees should be made aware of the social message they convey. Lack of professional appreciation is not only expressed by disrespectful treatment. Rather, the task itself contains a message. This might be counterintuitive for supervisors at first, since for them the delegated task is only one thing among many others. Therefore, it is all the more important to be sensitive to the opposite perspective; the person who receives the task. Nevertheless, creating awareness among trainees about what the teacher job and the curriculum include might as well be a step toward perceived legitimacy.

Regarding study seminars, the attendance of the seminar in general was perceived as especially illegitimate. A closer link to the school context might help here. Greater practical relevance in terms of a closer link to teaching and tailoring tasks to trainees' skills and experiences is necessary. The discussion of practical cases and worksheets and the planning of actual school lessons could be a way to reduce perceived illegitimacy. Moreover, inefficiencies should be avoided. A distancing from fixed structures and a loosening of guidelines might be useful. Instead, instructors and trainees could for example collectively decide on seminar contents and methods in order to get a better alignment with the needs and skills of a particular work group.

Increasing communication at school and providing more information as a signal of appreciation could result in significant improvement toward less illegitimacy at school. The establishment of standardized communication and information channels in terms of regular meetings to inform trainees about upcoming projects or classes could help to reduce perceived illegitimacy. Moreover, whenever an illegitimate task is not avoidable for some reason, appreciation could be expressed by at least explaining the situation.

Teaching trainees reported disrespectful treatment from pupils, parents, and supervisors. However, only supervisors were blamed for illegitimate tasks and more social support was only expected from them. They are the ones assigning tasks and are a major source of (direct and indirect) appreciation at work. Therefore, aside from improving task designs and framework conditions, supervisors are especially able to influence teaching trainees' perceptions of a situation in a positive manner. They are the people who should give social support to help trainees grow into their roles. Interventions in order to make supervisors aware of their role and the importance of appreciative task design, as well as interventions on how to give social support are promising approaches in the reduction of illegitimacy and are seen as important and necessary step toward an improvement of teaching trainees' health and well-being.

## Conclusion

The present work analyzed illegitimate tasks as an important aspect of the broader SOS-concept (Semmer et al., 2007) in the context of the German teaching trainee curriculum. Leading questions addressed teaching trainees' role perception and their experience of illegitimate tasks during the curriculum. The reported frequency of these tasks showed their relevance and applicability in the teaching trainee curriculum. Moreover, tasks were often considered illegitimate when no agreement existed whether they are located within a trainee's professional role or not. While unnecessary tasks often occurred in the study seminar, unreasonable tasks and related social circumstances such as lacking social support, lacking communication and disrespectful treatment were mostly prevalent in the school context. Although not included in the interview questions, reports by trainees about feeling stressed were an alarming hint at the negative consequences illegitimate tasks can evoke. Therefore, we recommend further research on illegitimate tasks in the teaching context with a special focus on the influence of organizational and situational factors to get a more in-depth understanding of the effects these tasks have on trainees' health and well-being.

## Ethics statement

The study was performed in consensus with requirements, including participants' information about their rights and guarantee of anonymity. All subjects gave informed consent in accordance with the Declaration of Helsinki.

## Author contributions

SF and MK designed the study. SF, KO, and MK structured the ideas and SF and HK did the analyses. All authors read and approved the final manuscript.

### Conflict of interest statement

The authors declare that the research was conducted in the absence of any commercial or financial relationships that could be construed as a potential conflict of interest.
